# Identifying Prevalence and Risk Factors for Intimate Partner Violence in Pregnant Women in Rural Guatemala

**DOI:** 10.26502/fjwhd.2644-28840066

**Published:** 2021-10-11

**Authors:** Anna E Lee, Claudia Rivera, Saskia Bunge Montes, Andrea Jimenez-Zambrano, Amy Nacht, Antonio Bolanos, Edwin Asturias, Stephen Berman, Gretchen Heinrichs, Margo S Harrison

**Affiliations:** 1 University of Colorado, School of Public Health, Center for Global Health, Colorado, USA; 2 Fundacion Integral por la Salud de los Guatemaltecos, Center for Human Development, Retalhuleu, Guatemala; 3 Department of Obstetrics and Gynecology, University of Colorado, School of Medicine, Colorado, USA; 4 Denver Health, Department of Obstetrics and Gynecology, Colorado, USA

**Keywords:** Intimate Partner Violence, Pregnancy, Food Insecurity, Guatemala, Latin America

## Abstract

**Background:**

Victims of intimate partner violence (IPV) during pregnancy experience significant physical and mental health consequences and adverse birth outcomes. Our objective was to describe the prevalence of IPV, and risk factors associated with IPV in pregnant, rural Guatemalan women.

**Methods:**

This retrospective cohort study was completed using quality improvement data gathered during routine prenatal health visits to women of Trifinio, Guatemala, by the Madres Sanas maternal health program from 2018 through 2020. Chi-square and t-tests were used to determine if there were differences in characteristics between women who self-reported experiencing IPV and those who did not. If differences occurred (p < 0.2), those covariates were included in a multivariable logistic regression to determine sociodemographic risk associated with IPV.

**Results:**

583 women were enrolled with Madres Sanas between October 10, 2018, and October 1, 2020, and reported on IPV. Nineteen (3.26%) women reported experiencing IPV. The highest prevalence of IPV (7.6%) occurred in the sub-group of women who experienced food insecurity during the past year. The sole covariate of all sociodemographic and health characteristics which differed significantly between women who reported experiencing and not experiencing IPV was food insecurity. A regression model found that those who had worried about ability to buy food in the past year had a 3.19-fold increase in the odds that they experienced IPV (95% CI 1.072, 9.486, p-value 0.037).

**Conclusion:**

Among this convenience sample of women, the prevalence of IPV was 3.26%. Food insecurity was associated with increased odds of experiencing IPV, highlighting an opportunity for interventions.

## 1. Introduction

Intimate partner violence (IPV) is the most common form of violence against women and is considered a global public health and human rights issue. IPV can manifest in many ways, including stalking, physical and/or sexual violence, and psychological and verbal abuse [1]. IPV impacts women’s health mentally and physically [1]. Women can suffer injuries due to physical violence, such as fractures, lacerations, head traumas, pain disorders, hypertension, urinary tract infections, and unintended pregnancies [1, 2]. Mental health impacts may include an increased risk of substance use, depression, suicidal thoughts, and posttraumatic stress disorder [1, 2]. The prevalence of IPV increases for pregnant women, with a higher associated risk of miscarriage, abruptio placenta, preterm delivery, perinatal mortality, fetal bruising, and low birthweight [2–4].

IPV is vastly underreported worldwide [1] and data on the prevalence of IPV may not be widely collected and can be difficult to find [5]. In the Americas, the prevalence of women experiencing IPV during their lifetime ranges from 14% in Brazil to 58% in Bolivia [5]. This large range of prevalence may be due to many factors, from reporting to social economic statuses. Guatemala, a country with high fertility rates, limited healthcare for rural inhabitants, and social and gender inequality [6], has a lifetime prevalence of IPV among women aged 15–49 of 21.2% [5]. The National Maternal and Child Health Survey (La Encuesta Nacional de Salud Materno Infantil (ENSMI)) in Guatemala found that the prevalence of a woman ever experiencing IPV was 24.7% in urban areas and 18.7% in rural areas from 2014 to 2015 [7]. Factors which may increase the risk of experiencing IPV during pregnancy in LMICs include a young age of the mother [8] as well as when a woman’s pregnancy is unplanned or un-wished for [5].

Much of the current research and proposed interventions to address IPV come from high-income countries like the United States or Europe raising the question of the validity of applying this work to LMICs. The research conducted in lower- or middle-income countries show that prevalence of IPV and its characteristics and risk factors may be quite different that those in places like Europe or the US [5]. This retrospective cohort study aimed to define the prevalence of IPV and associated risk factors in pregnant women in southwest Trifinio, a remote, rural region of Guatemala. We predicted that the prevalence of IPV in this rural population will be near 15%, and that younger women would have a higher prevalence of IPV during pregnancy. A better understanding of the prevalence and risk-factors for IPV in this convenience sample will aid and direct the development of site-specific interventions that can address IPV and reduce the risk of adverse outcomes for these women.

## 2. Methods

### 2.1 Background and setting

The University of Colorado’s Center for Global Health collaborated with Agroamerica, one of the largest employers in the southwest Trifinio region, to create the Center for Human Development (CHD). The goal of CHD was to improve the health and well-being of individuals in the region, by providing community-based medical care. The Trifinio region in the lowlands of Guatemala is comprised of 22 rural communities which have high rates of migrant workers, poverty, limited access to healthcare, and food insecurity. A consequence of these factors is high rates of maternal complications and poor pregnancy outcomes [9]. This inspired the CHD to create the Madres Sanas (MS) program, a project to improve maternal health using community nurses [9]. The MS program includes four prenatal visits during a patient’s pregnancy. During the nurse visits, trained nurses provide antenatal care and identify risk factors for pregnancy-related complications with subsequent referrals for higher level care. The MS nurses also collect demographic and health data from the enrolled women via mobile phone app-based questionnaires [9].

### 2.2 Study design and population

This study is a retrospective analysis of longitudinal, prospectively collected quality improvement data from the MS program from October 2018 to October 2021. The data belongs to the Center for Human Development of the Fundacion para la Salud Integral de los Guatemaltecos (FUNSALUD) and the database utilizes Research Electronic Data Capture (REDCap) via a secure e-health platform of the Center for Global Health. The primary purpose of the MS data collection is to assess program efficacy in improving pregnancy outcomes [9]. Women are recruited into MS from 12 of the 22 communities by community members, local leaders, community outreach nurses, and the health clinic [9]. Approximately 30% of women from the nearby 12 communities were enrolled in the program [9].

The primary outcome of interest was if the pregnant woman had ever experienced IPV in her lifetime. This was assessed with the question, “Has any man ever hit, kicked, or abused you physically or sexually?”. This question is asked during the first visit from MS nurses via questionnaire. Inclusion criteria for this analysis were that the individual 1) was registered with the MS program, 2) reported on whether they had or had not experienced IPV, and 3) continued their participation in the MS program through the post-partum period. We excluded data from women who did not report on experiencing IPV. After applying the exclusion criteria to the sample, the final analysis included 583 pregnant women.

### 2.3 Statistical analysis

The data were analyzed using SAS® OnDemand for Academics 3.8. A descriptive analysis was performed to determine prevalence of IPV, and to summarize the demographics of pregnant women who had experienced IPV. A multivariate logistic regression was applied to find the odds of experiencing IPV with different potential sociodemographic and health risk factors. In the logistic regression model, variables were collapsed into dichotomous categories, with the categories being informed by the literature. The following variables were defined and included in our analysis. Community distance from the CHD (with “near” meaning the community borders the CHD, and “far” meaning another community stood between it and the CHD). Maternal age was made dichotomous by splitting women into the groups less than 19 years of age and 19 years and older. Coupled status was defined as whether they were in a relationship (“coupled”) or not. Weekly income was separated into making 1000 quetzal or less, or more than 1000 quetzal. Education level was split into having completed up to a 10^th^ grade level of education or above that level. Reading ability, whether birth control was used prior to pregnancy, whether they were employed by Agroamerica, and whether a referral for specialized care was needed during their pregnancy were split into their respective answers of “yes” and “no”.

Food insecurity was assessed with two questions and yes/no responses; 1) “In the past year, have you been worried that you wouldn’t have enough money to buy food?” and 2) “In the past year, did the food you bought run out and you no longer had money to buy more?”. The number of previous pregnancies was split into whether the woman had one or fewer previous pregnancies or more than one. Variables that were missing > 10% of data were excluded from this analysis.

In bivariate comparisons we analyzed characteristics using Fisher’s exact test due to low cell sizes, and Pearson’s chi-squared when cell size permitted. Variables with a p ≤ 0.2 were considered in a multivariable regression model. Testing for interactions was completed using chi-squared tests and −2log likelihood tests, and no evidence of variable interaction was found.

## 3. Results

The descriptive statistics shown in Table 1 reveal some notable features about our convenience sample. Of the 583 pregnant women in our convenience sample, 19 answered “yes” to the question asking about IPV, making the overall prevalence of IPV 3.3%. The prevalence of IPV in women 19 years old or younger was 2.8%, and 3.3% in women over 19 years old. The highest prevalence of IPV was in women in the sociodemographic subgroups of those who could not read (8.1%), those women who were worried they would not be able to buy enough food in the past year (7.6%), and those who ran out of food after buying it in the past year (7.4%). Otherwise, the prevalence of IPV throughout the examined subgroups ranged from 0.0% (in women who had above a grade ten education) to 8.1% (women who can not read). Bivariate comparisons found that the following had a p-value of less than or equal to 0.05: ability to read (p=0.018), food insecurity (women worried about ability to buy enough food) (p=0.005), and number of previous pregnancies (p=0.022). The comparison found the following variables had a with a p-value of ≤0.2: coupled status, ability to read, both food insecurity variables, and number of prior pregnancies. The multivariate regression including variables with a p-value of ≤0.2 showed that only the first food insecurity variable (women who worried they would not be able to buy enough food in the past year) was found to be significant (with a p-value of <0.05) (Table 2). The associated odds ratio with this variable was 3.190 (95% confidence interval: 1.072, 9.486) with a p-value from the maximum likelihood analysis of 0.037.

## 4. Discussion

We hypothesized that the prevalence of IPV in pregnant women in our cohort would be 15%. It was found to be much lower (3.3%), with the highest prevalence being found in the subgroups of women who can’t read and those with food insecurity. We also hypothesized that pregnant women who were younger would experience a higher prevalence of IPV, and this was found to be incorrect. Women who were 19 years and older experience a prevalence of 3.3%, 0.5% higher than in women who are younger than 19 years old. This study also aimed to define what risk factors, if any, were associated with IPV for these pregnant women. We found that the only variable associated with an increased odds of IPV was related to food insecurity; women who, in the past year, worried that they wouldn’t be able to buy an adequate amount of food with their income. In this case, we found that food insecurity was associated with a 3.19-fold increase in the odds that they have ever experienced IPV (95% CI 1.072, 9.486).

The results of our analysis were surprising. The prevalence of IPV in this cohort of women is much lower than predicted, and several factors may contribute to this. IPV is notoriously underreported to begin with and might be even more underreported in this rural population. Research has shown that barriers to reporting IPV include personal discomfort with the issue, lack of knowledge, time constraints, and a lack of provider-patient relationship [10, 11]. Any of these factors may be present for women in our cohort. The question about IPV asked by the MS nurses in the MS questionnaire does not have a comprehensive definition of IPV, and places most of the emphasis on physical abuse. This could mean that some women do not think their experience of IPV fits into that definition, and thus do not report it. Underreporting may also be due to the fact that this question is only asked once, at the very first visit by MS, and never revisited. A women might not have built enough of a relationship with the MS nurses to feel comfortable answering the question at that time, and then is not asked again. Looking to the Guatemalan ENSMI 2014–2015, we see that a much higher reported prevalence of IPV in rural areas (18.7%) was found [7]. The ENSMI may have gotten more responses for several reasons; 1) consent was obtained from the surveyed woman to talk about abuse, and the strictest confidentiality guidelines were explained to participating women and were followed during and after the interview [7] and 2) questions about abuse were split into broadly defined categories of sexual violence and physical violence [7]. These elements of the data collection would be Finally, another aspect that could add to underreporting is the fact that the woman’s partner or other family members might be there while the woman fills out the questionnaire. Although the questions are not necessarily read out loud to the woman (unless of course she cannot read), a woman may still not feel comfortable answering the question even in written form if her partner is near (even if the IPV was not perpetrated by them). There may also be a lack of motivation to report IPV because the woman sees no recourse from it, especially if resources are not presented to her.

The finding that pregnant women older than 19 years of age had a higher prevalence of IPV than woman 19 years old and younger was also contrary to our hypothesis. We expected younger pregnant women to have a higher prevalence of IPV than their older counterparts. This result may also have something to do with how the question is worded. Because the question asks about ever experiencing IPV, it is possible that those women who are older may have experienced IPV more. To address the question of whether younger women experience a higher prevalence of IPV during pregnancy would require data with more refined questions, such as “Are you currently experiencing IPV?” or “If you have previously experienced IPV, when?”. Our finding that the only significant risk factor associated with IPV was food insecurity was also unexpected. For women of comparable backgrounds to those in our cohort, factors such as education level and age were found to be risk factors [12]. This cohort of women is from a very poor region in Guatemala, where food insecurity is high. If a household has worried in the past year about being able to afford food to feed their family, this may create an environment which may be conducive to IPV. Studies have also demonstrated that there is a strong positive association between food insecurity and IPV [12, 13]. Food insecurity in this group might be a predictable indicator about stability in the home and opens opportunities for interventions to address both food insecurity and IPV.

Some limitations of our research include the possibility of self-selection bias. Participants enroll in the Madres Sanas program and complete surveys voluntarily, meaning they may choose not to answer some. The data on IPV are also limited, as is it only asked once, and does not use a comprehensive definition of IPV. Reporting and misclassification biases may also be introduced as the women are self-reporting IPV. Due to the study using a small population of migrant, rural Guatemalan women reporting through quality improvement data, another limitation is that non-generalizability is introduced.

A strength of this study is that this analysis provides baseline data that can be used to generate further research questions or studies in a population where IPV is poorly studied. Another strength of the study is the quality of the data as the data is from an established quality improvement database that regularly undergoes quality assurance reviews. These findings are also valuable to the CHD and will help guide the development of interventions aimed at reducing IPV for pregnant women. Interventions addressing food insecurity in the population should be explored. These may involve foodbanks or meal programs. Food insecurity in the population should also be further studied to better understand the association with IPV. This research also highlights that the data collection process on IPV needs to be improved. The definition of IPV should encompass all types of IPV, not only the physical aspects. Elements of data collection that ENSMI 2015 employed (obtaining consent, ensuring strict confidentiality, and asking several questions about different types of IPV) should perhaps be mirrored to improve reporting.

Additionally, data on IPV should be collected at every Madres Sanas visit. Questions that address when IPV occurred for the women should also be incorporated to better understand the scope of IPV in this population. Education on IPV would also be beneficial for women of this population so they are better equipped to answer these questions and may improve underreporting of IPV. Education on community resources that may exist for those who have experienced IPV should also be included in any education on IPV.

In conclusion, our analysis of the data show that the prevalence of IPV in pregnancy, rural Guatemalan women who enrolled in the Madres Sanas care program in southwest Trifinio between October 10, 2018 and October 1, 2020 had an IPV prevalence of 3.3%, and that the subgroup with the highest prevalence of IPV were those women who could not read and those who experienced food insecurity. The only sociodemographic characteristic associated with greater odds of IPV was food insecurity. While prevalence is lower than expected based on baseline data in Guatemala, this is most likely due to underreporting, and better understanding the population-specific prevalence of IPV is vital. Future steps include assessing and improving the resources available to women who have experienced IPV in the population, and then disseminating this information. Further research into food insecurity and associated variables should also be conducted to better understand how to improve outcomes for women who are exposed to the risk-factor of food insecurity as well.

## Figures and Tables

**Table 1:** Maternal sociodemographic/health descriptive statistics of pregnant Guatemalan women by self-reported experience of IPV and associated prevalence of IPV.

	Reported abuse (n and %)	No reported abuse (n and %)	Total	Prevalence (%)	p-value

Total women	19 (3.26)	564 (96.74)	583	3.3	

From community:					
• Near	9 (1.55)	320 (54.98)	329 (56.53)	2.8	0.413
• Far/other	10 (1.72)	243 (41.75)	253 (43.47)	4.0	
*n missing = 1*					

Maternal Age (y)					
• ≤ 19	4 (0.70)	141 (24.69)	145 (25.39)	2.8	0.213
• >19	14 (2.45)	412 (72.15)	426 (74.61)	3.3	
*n missing = 12*					

Coupled					
• Yes	15 (2.57)	501 (85.93)	516 (88.51)	2.9	0.108
• No	4 (0.69)	63 (10.81)	67 (11.49)	6.0	

Weekly income					
• <− 1000	16 (2.99)	503 (93.84)	519 (96.83)	3.1	0.329
• >1000	1 (0.19)	16 (2.99)	17 (3.17)	5.9	
*n missing = 47*					

Education level					
• up to 10	19 (3.28)	520 (89.66)	539 (92.93)	3.5	0.243
• > 10	0	41 (7.07)	41 (7.07)	0.0	
*n missing = 3*					

Reading?					
• Yes	13 (2.20)	493 (85.00)	506 (87.24)	2.6	0.018
• No	6 (1.03)	68 (11.72)	74 (12.76)	8.1	
*n missing = 3*					

Used birth control prior to pregnancy					
• Yes	5 (0.86)	134 (23.14)	139 (24.01)	3.6	0.202
• No	14 (2.42)	426 (73.58)	440 (75.99)	3.2	
*n missing = 4*					

Works for Agroamerica?					
• Yes	1 (0.17)	19 (3.28)	20 (3.45)	5.0	0.356
• No	18 (3.11)	541 (93.44)	559 (96.55)	3.2	
*n missing = 4*					

Worried wouldn’t be able to buy enough food?				7.6	
• Yes	9 (1.55)	109 (18.79)	118 (20.34)	2.2	0.005
• No	10 (1.72)	452 (77.93)	462 (79.66)		
*n missing = 3*					

Run out of food in past year?					
• Yes	4 (0.69)	50 (8.64)	54 (9.33)	7.4	0.066
• No	15 (2.59)	510 (88.08)	525 (90.67)	2.9	
*n missing = 4*					

Number of previous pregnancies				1.6	
• 1 or fewer	5 (0.88)	304 (53.24)	309 (54.12)	5.0	0.022
• More than 1	13 (2.28)	249 (43.61)	262 (45.88)		
*n missing = 12*					

Needed referral during care?					
• Yes	2 (0.34)	67 (11.49)	69 (11.84)	2.9	0.213
• No	17 (2.92)	497 (85.25)	514 (88.16)	3.3	

p-value found using Fisher’s Exact Test, except for “community”, where a Pearson’s chi-square test was used

**Table 2:** ORs for multivariate logistic regression model including variables that had p<0.2 in bivariate comparisons.

Variable	Odds ratio	95% confidence interval	P-value (maximum likelihood analysis)
Coupled status			
Reading level	2.501	0.866, 7.223	0.09
Food insecurity 1	3.190	1.072, 9.486	0.037
Food insecurity 2	1.098	0.288, 4.183	0.892
Prior pregnancies	2.637	0.893, 7.791	0.079

**Table 3:** Analysis of Maximum Likelihood Estimates: Interaction Testing Model Between Coupled and Reading Variables.

Parameter	Estimate	Standard Error	Wald Chi-Square	P-value
Coupled status	−1.065	0.931	1.307	0.253
Reading level	−1.649	1.058	2.429	0.119
Coupled*Reading	0.632	1.216	0.269	0.604
